# Supplementary material for: CRISPR/Cas9-Mediated SlMYBS2 Mutagenesis Reduces Tomato Resistance to Phytophthora infestans
Source: Int J Mol Sci. 2021 Oct 22;22(21):11423. doi: 10.3390/ijms222111423 (PMC8583707; doi:10.3390/ijms222111423)
Supplement: Supplementary file 1 [file ijms-22-11423-s001.zip › ijms-1412853-supplementary.pdf]

International Journal of
Molecular Sciences

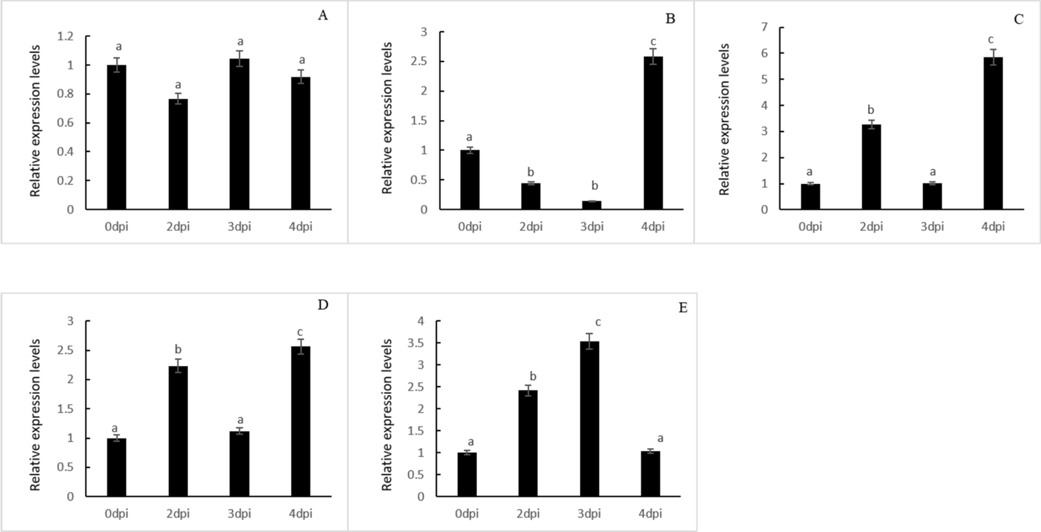

Figure S1. qRT-PCR analysis of five MYB family genes induced by late blight. It can be seen from the figure that only *SlMYBS2* meets the induction of pathogens. A:MYB28 B:MYB75 C:ANT1 D:MYB114 E: SlMYBS2.

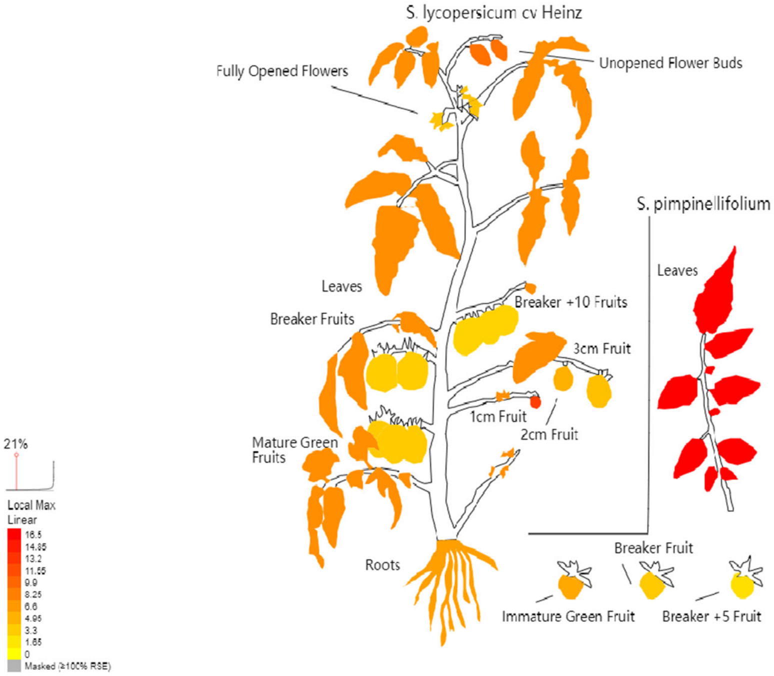

Figure S2. Expression pattern of SlMYBS2 in different tissues and organs. This image was retrieved from the public tomato eFP Browser tool. The red arrow indicates the preferential expression of *SlMYBS2* in leaves. A color key of expression values was shown at the left bottom comer.

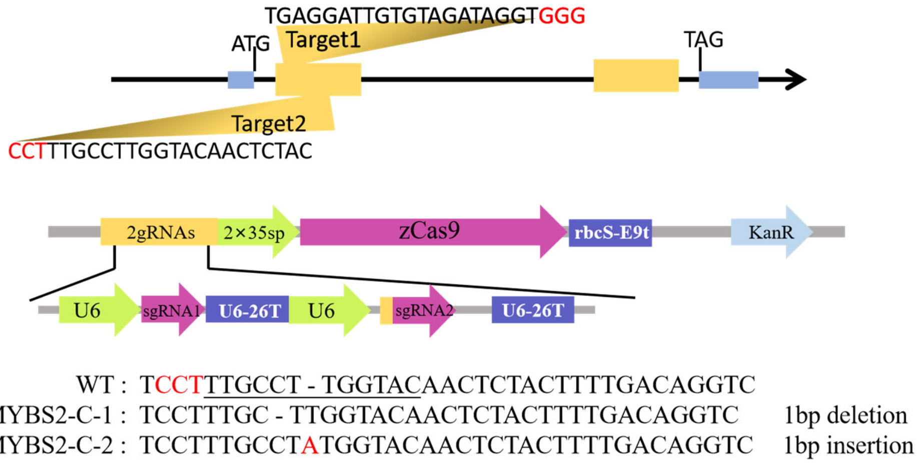

Figure S3. CRISPR/Cas9 induced mutations in the *SlMYBS2* gene. (A) A schematic map of the gRNA target sites on the genomic regions of SlHY5. Intergenic region and introns are shown as lines; exons are shown as yellow boxes; 3′UTR and 5′UTR are shown as blue boxes. The PAM motifs (NGG) are shown in red. (B) Schematic presentation of the T-DNA structure in CRISPR/Cas9-mediated genome editing construct. (C) Sequencing results of the *SlMYBS2* homozygous mutant lines from the T1 generation. The target sequence is underlined, the insertions are highlighted in red, and the dashes indicate deletions.

Table S1. *qRT-PCR.*

qRT-PCR	
CAT-F	GAAGCCAAATCCTAAGTCCC
CAT-R	GCCTGTAATCTTGTGGAATACC
SOD-F	CCAGAAGCATCATCAGACTTAC
SOD-R	GGGCAAGGTTATTCCAGA
APX-F	CCATTAGGGAGCAGTTTCC
APX-R	CCTTCAACAGGTGGTTCTG
PR1-F	GGGAGAAGCCAAACTACAAC
PR1-R	GAAATGAACCACCATCCG
PR2-F	TCCAGGTAGAGACAGTGGTAAA
PR2-R	CCTAAATATGTCGCGGTTGAGA
PR3-F	GAACGAGCTGGACAAGGTATT
PR3-R	CGTTGTGGCATGATGGTTTATT
PR5-F	CCCAAACACCCTAGCTGAAT
PR5-R	GGGCGAAAGTCATCGGTATATTA
SlMYBS2-F	ATAGTGAATTTCAAGAATGACAGTAGA
SlMYBS2-R	CAGCGTTACCCGACCCT
ACTIN-F	CGAGCAGTGTTTCCCAGTATT
ACTIN-R	AGCCTGGATAGCAACATACATAG
Cloning of CDS	
CDS-F	ATGACAGTAGATAAATCAAGAAGCTCAA
CDS-R	TCAAGGTTGGTCGTAGTAATATCCC
CRISPR/Cas9	
*SlMYBS2*gRT1	TGAGGATTGTGTAGATAGGTGTTTTAGAGCTAGAAAT
*SlMYBS2*AtU3bT1	ACCTATCTACACAATCCTCAGACCAATGTTGCTCC
*SlMYBS2*gRT2	GTAGAGTTGTACCAAGGCAAGTTTTAGAGCTAGAAAT
*SlMYBS2*AtU3dT2	GTAGAGTTGTACCAAGGCAAGACCAATGGTGCTTTG
